# Entropy-based selection for maternal-fetal genotype incompatibility with application to preterm prelabor rupture of membranes

**DOI:** 10.1186/1471-2156-15-66

**Published:** 2014-06-10

**Authors:** Shaoyu Li, Yuehua Cui, Roberto Romero

**Affiliations:** 1 Department of Biostatistics, St Jude Children’s Research Hospital, 262 Danny Thomas Place, Memphis, USA; 2 Department of Statistics and Probability, Michigan State University, Wells Hall, East Lansing, USA; 3 Division of Medical Statistics, School of Public Health, Shanxi Medical Universiy, 030001 Taiyuan, Shanxi, China; 4 Perinatology Research Branch, NICHD/NIH/DHHS, Bethesda and Detroit, USA; 5 Department of Obstetrics and Gynecology, University of Michigan, Ann Arbor, USA; 6 Department of Epidemiology and Biostatistics, Michigan State University, East Lansing, USA

**Keywords:** Complex disease, Pregnancy complications, Association study, Maternal-fetal genotype incompatibility

## Abstract

**Background:**

Maternal-fetal genotype incompatibility (MFGI) is increasingly reported to influence human diseases, especially pregnancy-related complications. In practice, it is challenging to identify the ideal incompatibility model for analysis, since the true MFGI mechanism is generally unknown. The underlying MFGI mechanism for different genetic variants can vary, and to use a single incompatibility model for all circumstances would cause power loss in testing MFGI.

**Results:**

In this article, we propose a practical 2-step procedure that incorporates a model selection strategy based on an entropy measurement to select the most appropriate MFGI model represented by data and test the significance of the MFGI effect using the chosen model within the generalized linear regression framework.

**Conclusions:**

Our simulation studies show that the proposed two-step procedure controls the type I error rate and increase the testing power under various scenarios. In a real data application, our analysis reveals genes having an MFGI effect, which may not be detected with a non-model selection counterpart.

## Background

Current advances in high-throughput biotechnology have popularized genome-wide association studies (GWAS) to detect genetic variants that increase the risk of complex diseases. Over the past decade, thousands of single nucleotide polymorphisms (SNPs) have been reported to be associated with various human diseases. Despite the numerous successes of GWAS, the majority of heritability for many complex diseases remains unexplained [1-5]. Recent genomic research provides compelling evidence that the cause of complex human diseases is multifactorial and involves both genetic and environmental factors. The lack of consideration of sophisticated components like gene-gene interactions, gene-environment interactions, and epigenetic functions can lead to the missing heritability for most common diseases.

The underlying genetic architecture can be especially complicated for diseases developed during human pregnancy, since both maternal and fetal genomes are involved. In general, the fetus inherits one copy of the genome from each of its parents, and the two copies are not identical. Previous family-based or twin studies indicated that the heritability for obstetric diseases is high. For example, it is reported in an earlier twins study that heritability was 17*%* for preterm delivery in first pregnancy and 27*%* for preterm delivery in any pregnancy [6] and heritability range of 25%–40% was suggested for birthweight and gestational length in another study [7]. Maternal and fetal genes, either individually or in combination, could increase the risk of diseases such as hemolytic disease of the newborn [8], preterm birth [9,10], small for gestational age [11], pre-eclampsia [12-14], and preterm prelabor rupture of membranes (pPROM) [15]. The incompatibility between maternal and fetal genotypes, in which the expression of genes from two generations lead to an opposite effect, plays a vital role and can increase the risk of these diseases. However, most current association studies on obstetric diseases have primarily focused on only one genome for susceptibility genomic loci; that is only the maternal or fetal genome was searched for associated genetic factors when a maternal or fetal disorder was studied.

Evidence support the important role of interaction between maternal and fetal genes, more than maternal genes alone for the etiology of pregnancy complications, are accumulating [16-19]. In other words, an increased risk of certain disorders could be due to a specific combination of maternal and fetal genotypes. The mother and fetus share only one allele. Mismatches between maternal and fetal genotypes may lead to adverse effects when a fetus resides in utero and increase the risk of disease. A good example of this deleterious effect comes from the allogenic response. If a bi-allelic locus has a null allele and an antigen-coding allele, the mother is homozygous for the null allele, and the fetus inherits an allele from the father which codes for an antigen, the mother may produce an allogenic response to the fetal antigen, which is harmful to the fetus. This type of incompatibility between maternal and fetal genotypes is well illustrated by Rh incompatibility, which is developed when a pregnant woman is Rh-negative (d/d) and the fetus is Rh-positive (D/d) in the RhD locus. Red blood cells from the fetus can cross into the maternal blood stream through the placenta. The maternal immune system treats Rh-positive fetal cells as external attacks and makes antibodies against the fetal blood cells. These antibodies may cross back into the developing fetus and destroy its circulating blood cells, which can cause hemolytic disease of the newborn (HDN). Therefore, the identification of genes with maternal-fetal genotype incompatibility (MFGI) by searching parental and offspring genomes simultaneously is highly recommended [20-26].

Study designs in which data are collected from parent-offspring triads or mother-offspring dyads are the most commonly used to investigate the marginal and joint effects of maternal and fetal genes. Most currently available statistical approaches for analyzing this type of data fall in the framework of generalized linear regression models. Maternal fetal genotype tests based on the log-linear modeling for child-parent triads have been developed [20,27-29]. These tests are robust to population stratification because they compare the distribution of affected and unaffected individuals given the parental mating type instead of comparing frequencies of alleles/genotypes between cases and controls. However, these tests and their extensions require at least some paternal data are available. For situations when paternal data are 100*%* missing, the dyad sampling data, methods based on logistic regression models were proposed [22,23,25].

Although it has been widely hypothesized that mismatches between maternal and fetal genotypes can cause incompatibility, the underlying biological mechanism remains unclear. Therefore, it is challenging to appropriately model incompatibility and code the corresponding variable accordingly. That is, suppose a variable *G*_*i* *c*_ denotes the MFGI effect, it is problematic to decide when to code the variable as 1 or 0. Parimi et al. [30] evaluated the performance of 6 plausible incompatibility models and concluded that the most comprehensive model, which codes genotype incompatibility whenever maternal and fetal genotypes are different, consistently outperformed other models. However, only the maternal-fetal incompatibility effect was simulated in their study, and the maternal main effect and the fetal main effect were not considered along with MFGI. When a maternal or fetal main effect co-exists with MFGI, this approach dramatically inflates the type I error. Even if only an incompatibility effect is present, the recommended model does not always achieve greater power than the true incompatibility models.

In this study, we developed a 2-step statistical strategy for testing MFGI effects in designs that collect data from the mother and offspring that can increase the testing power under a wide range of scenarios. We propose to select the MFGI model based on an entropy measurement via a permutation procedure; then we test the MFGI effect using the selected incompatibility model within the logistic regression framework.

## Methods

### Genetic model

Consider a study that enrolls case and control mother-offspring pairs from a target population. Collected data include genotypes of mothers and offspring, disease phenotype (phenotype of mother or child) of interest, and other covariates with a total of *n* independent mother-offspring pairs (*n*_0 _controls and *n*_1 _cases, *n*_1 _+ *n*_0 _= *n*). Let *G*_*m *_and *G*_*o *_denote the maternal and fetal genotypes of a particular SNP, respectively. Under the commonly used additive genetic model, *G*_*m*/*o *_= 0,1, or 2 if the mother/offspring has 0, 1, or 2 copies of the minor allele. Let *Y* = (*y*_1_,*y*_2_,⋯,*y*_*n*_)^*T *^denote the vector of the phenotype, where *y*_*i *_is the dichotomous disease outcome of the *i*^*th *^family unit in the sample, in which *y*_*i *_= 1 or 0 corresponds to the affected or unaffected individuals.

Consider a bi-allelic genomic locus with 2 alleles: A and a, where A denotes the rare allele. Following the Mendelian inheritance, there are seven possible maternal-fetal genotype combinations (see Table 1). The 4 mismatched maternal-fetal genotype combinations are denoted as *M*_1_,*M*_2_,*M*_3_, and *M*_4_. It is possible that any of the mismatched maternal-fetal genotype combination leads to incompatibility or that only a specific mismatched genotype combination or a certain collection of these genotype combinations is associated with the risk of disease.Therefore, in the absence of evidence from molecular genetics analysis, it is challenging to determine which incompatibility model fits the biological mechanism. Here, we consider 11 biologically-plausible incompatibility models(Table 2) and propose a 2-step procedure to identify genomic loci that have a MFGI effect on a disease outcome of interest. We first select an MFGI model based on an entropy measurement and then test the statistical significance of MFGI using the chosen incompatibility model. Details of the 2-step procedure are described in the following section.

**Table 1:** Possible maternal-fetal genotype combinations

	***G***_***o***_		
***G***_***m***_	***A A *** **(2)**	***A a *** **(1)**	***a a *** **(0)**
*AA*(2)	0	*M*_1_	-
*Aa*(1)	*M*_2_	0	*M*_3_
*aa*(0)	-	*M*_4_	0

**Table 2:** Biologically plausible models of maternal-fetal genotype incompatibility

**Model**	**GC**	***G***_***m***_	***G***_***o***_	**Scenario**
1	*M*_1_	*AA*	*Aa*	Mother has 1 more copy of allele *A* than the heterozygous offspring
2	*M*_2_	*Aa*	*AA*	Offspring has 1 more copy of allele *A* than the heterozygous mother
3	*M*_3_	*Aa*	*aa*	Mother has risk allele *A* that the offspring does not
4	*M*_4_	*aa*	*Aa*	Offspring has risk allele *A* that the mother does not
5	*M*_1_	*AA*	*Aa*	Mother-offspring pair has 3 copies of *A* allele
	*M*_2_	*Aa*	*AA*	
6	*M*_1_	*AA*	*Aa*	Mother has 1 more copy of *A* allele
	*M*_3_	*Aa*	*aa*	
7	*M*_1_	*AA*	*Aa*	Offspring has an allele that the mother does not
	*M*_4_	*aa*	*Aa*	
8	*M*_2_	*Aa*	*AA*	Mother has an allele that the offspring does not
	*M*_3_	*Aa*	*aa*	
9	*M*_2_	*Aa*	*AA*	Offspring has 1 more copy of the *A* allele
	*M*_4_	*aa*	*Aa*	
10	*M*_3_	*Aa*	*aa*	Mother-offspring pair possesses 3 copies of allele *a*
	*M*_4_	*aa*	*Aa*	
11	*M*_1_	*AA*	*Aa*	All possible mismatched maternal-fetal genotype combinations
	*M*_2_	*Aa*	*AA*	
	*M*_3_	*Aa*	*aa*	
	*M*_4_	*aa*	*Aa*	

A: Minor allele;

GC: Genotype combination.

### Statistical model

The information theory, which was initially developed in the 1940s [31] to quantify the transmission of information in communication channels within a rigorous mathematical framework, has gained much attention in genetic association studies in recent years [32-35]. Our aim is to propose a model selection strategy to choose the MFGI model best represented by the data using the entropy theory. Before introducing the model selection strategy, we discuss some basic concepts about the information theory. Entropy measures the uncertainty of a random variable. For a discrete random variable *X*, entropy is defined as:

(1) $$H(X)=-\overset{d}{\underset{i=1}{\sum }}P(X=x_{i}){\text{log}}_{b}P(X=x_{i})$$

where *x*_*i*_,*P *(*X *= *x*_*i*_),*i *= 1,2,⋯,*d* are the possible values of *X* and their corresponding probabilities; *b* is the base of the logarithm and is commonly assumed to be 2 in the information theory. We propose the following 2-step procedure to test MFGI effects: **Step 1: Select the MFGI model** Let *p* and 1 - *p* be proportions of cases and controls, respectively, in a given data set. Entropy of the disease outcome can be computed

(2) $$H(D)=-{\text{plog}}_{2}(p)-(1-p){\text{log}}_{2}(1-p)$$

This entropy serves as a measure of the uncertainty of disease outcome in the initial data set.

Under each of the 11 plausible MFGI models listed in Table 2, the mother or offspring can be characterized as “high risk” or “low risk” based on their genotype combinations. For example, under Model 1, mother-offspring pairs with genotype combination *M*_1 _= (*AA*,*Aa*) are considered “high risk” and other combinations are considered “low risk”. The high and low risk labels split the initial data set into 2 subsets. Entropy of disease outcome within each subset, *H *(*D*|*risk *= *high*) and *H *(*D*|*risk *=*low*), can be calculated using Equation (2). The conditional entropy of disease status, given a particular MFGI model, is then defined as

(3) $$\begin{array}{lll} H(D|\text{MFGI}) & =H(D|\text{risk}=\text{high})P(\text{risk}=\text{high})\; & \\ & \;+H(D|\text{risk}=\text{low})P(\text{risk}=\text{low})\; \end{array}$$

This conditional entropy measures the remaining amount of uncertainty of disease outcome given the MFGI model. The difference between this conditional entropy and the original entropy is the information gain (or mutual information), which reflects the amount of information that a certain MFGI model provides (Equation (4)).

(4) IG(D;MFGI)=H(D)-H(D|MFGI)

To adjust for the uncertainty of disease status due to sampling, the information gain ratio was used (Equation (5)) as the criterion to select the optimal model to code the MFGI effect.

(5) $$R=\text{IG}(D;\text{MFGI})/H(D)=1-\frac{H(D|\text{MFGI})}{H(D)}$$

As shown in Table 2, Model 11 is the most comprehensive model because it includes all 4 incompatible maternal-fetal genotype combinations. The study by Parimi et al. (2008) recommends this model as “optimal” when decoding the MFGI effect. Herein we consider this model as the default model. The information gain ratio was calculated for each of the 11 plausible MFGI models and, then we selected the model that has the largest information gain ratio as the candidate model. Since a candidate model could be chosen by chance and does not reflect the real functional mechanism, a permutation procedure is used to assess how likely the candidate model will be chosen under the assumption of no genetic association as follows: 

1. Obtain the information gain ratio {*R*_*i*_,*i *= 1,2,⋯,11} for each model and identify the model with the maximum information gain ratio *R*^*max *^= *max *{*R*_1_,*R*_2_,⋯,*R*_11_} as a candidate model;

2. For *b *= 1,2,⋯,*B*, permute the disease label and obtain the maximum information gain ratio $R_{b}^{\text{max}}=\text{max}\{R_{1,b},R_{2,b},\cdots \;,R_{11,b}\}$;

3. Calculate the empirical p-value of selecting the model by chance

$$p-\text{value}=\frac{1}{B}\overset{B}{\underset{b=1}{\sum }}I(R_{b}^{\text{max}}>R^{\text{max}})$$

If the obtained empirical P-value is less than a pre-defined cutoff *τ* (say *τ *= 0.0001), we can conclude that the candidate model was not selected by chance and will be used as the analysis model in the next step of testing. Otherwise, Model 11 will be used as the analysis model. **Step 2: Test the MFGI effect** Once an optimal incompatibility model is selected, it will be used to code the incompatibility effect in a logistic regression model to assess the significance of the incompatibility effect, that is,

(6) $$\text{logit}P(Y=1|G_{m},G_{o})=\beta +\beta_{m}G_{m}+\beta_{o}G_{o}+\beta_{\text{ic}}G_{\text{ic}}$$

where *G*_*m *_and *G*_*o *_represent the maternal and offspring additive variables, respectively, which are coded as 0, 1, or 2 corresponding to aa, Aa, and AA, respectively, where A is the risk allele; and *G*_*ic *_is the variable of MFGI. The value of *G*_*ic *_depends on the selection result from Step 1. For example, if Model 1 is selected as the analysis model, then *G*_*ic *_= 1 for mother-offspring pairs with genotype combination (*AA*,*Aa*) and *G*_*ic *_= 0 otherwise. Testing the MFGI effect corresponds to testing the null hypothesis *H*_0_:*β*_*ic *_= 0. The likelihood ratio test was applied for this purpose.

## Simulation

To demonstrate that the proposed approach is valid in controlling the type I error rate and that it is statistically powerful, we conducted a series of simulations under the null and alternative hypotheses. Genotypes of *N *= 1,000,000 families (parents and a child) were generated in a population assuming symmetric mating and Mendelian transmission of alleles. Parental genotypes were generated by multinomial distribution with a pre-specified genotype frequency. Either the Hardy-Weinberg equilibrium (HWE: minor allele frequency = 0.2) or the Hardy-Weinberg disequilibrium (HWD: genotype frequency =(0.18,0.47,0.35) for homozygous carriers, heterozygotes, and noncarriers of the minor allele, respectively) was assumed. Fetal genotypes were simulated based on parents’ genotypes following Mendelian inheritance. Paternal data were then dropped to mimic the maternal-fetal study design. Binary phenotypes were simulated based on a quantitative liability variable *Z*=(*z*_1_,*z*_2_,⋯,*z*_*N*_)^*T*^, where *z*_*i *_denotes the liability variable of the *i*^*th *^subject. A threshold was determined to ensure that disease prevalence remained at 5*%*. Mother-offspring pairs with the underlying quantitative liability that exceeded the threshold were “diagnosed” as affected and others as unaffected. Simulated data were treated as a population. Then samples with the size *n* were randomly taken for subsequent analysis.

The underlying quantitative liability trait was simulated through the following regression model (Equation (7)),

(7) $$z=\alpha +\alpha_{m}G_{m}+\alpha_{o}G_{o}+\alpha_{\text{ic}}G_{\text{ic}}+\varepsilon$$

where *α*s are defined the same way as *β*s in Equation (6). Without loss of generality, we set the overall mean *α *= 0 and *σ*^2 ^= 1. Performance of our proposed two-step approach (called the model selection approach) was compared with that of its non-model selection counterpart (called the full model approach). Quantitative data were generated using a particular MFGI model listed in Table 2, called the data generating model. Various scenarios were considered (Table 3): Scenario I assumes no genetic effect at all; Scenarios II and III generate data under the null hypothesis of no MFGI effect while allowing maternal or fetal main effect; Scenarios IV-VI simulate the MFGI effect along with maternal and/or fetal main effects; and Scenarios VII-IX assume the MFGI effect only at 3 different heritability levels (*h*^2 ^= 0.05,0.10,0.15). The effect size of incompatibility was computed as described by Parimi et al.: let $\sigma_{T}^{2}=\alpha_{\text{ic}}^{2}q(1-q)+\sigma^{2}$ where *q* is the proportion of incompatible maternal-fetal genotypes in the simulated population. For a given heritability level *h*^2^, we can calculate the incompatibility effect through the equation $h^{2}=1-\sigma^{2}/\sigma_{T}^{2}$.

**Table 3:** Simulation scenarios with different parameter values

**Scenarios**	**I**	**II**	**III**	**IV**	**V**	**VI**	**VII**	**VIII**	**IX**
*β*_*m*_	0	0.4	0	0.4	0	0.2			
*β*_*o*_	0	0	0.4	0	0.4	0.2			
*β*_*i* *c*_	0	0	0	0.4	0.4	0.4			
*h*^2^							0.05	0.10	0.15

## A case study

We illustrated the proposed method via an application to a sub-analysis of a broader candidate gene study that investigates the role of genetic factors on the risk of complications of pregnancy. Details of this sub-study have been previously published in a genetic association study [15]. Briefly, this case-control study includes patients with preterm prelabor rupture of membranes (pPROM) and their neonates and control mothers with a normal pregnancy and their neonates. Patients of Hispanic origin were enrolled in a research protocol at the Sotero del Rio Hospital, Santiago, Chile.

pPROM occurs in 3%–4.5% of pregnancies in the United States and is responsible for about 30*%* of preterm births [15]. Although previous studies have suggested the presence of predisposing genetic factors for pPROM [9,10,36,37], the underlying genetic architecture remains unclear. SNPs in 190 candidate genes were selected and genotyped based on their possible biological roles in obstetrical diseases. We analyzed phenotypic and genotype data from the study to determine whether incompatibilities between the maternal and fetal genotypes increase the risk of pPROM. Six samples were removed because of large proportion of missing genotypes (> 50*%*) in either the mother sample or the offspring sample. Also, when searching across SNP markers, samples that did not follow Mendelian inheritance were excluded from the analysis. Our analysis included 742 SNPs in 190 candidate genes for 721 mother-offspring pairs (case-control ratio = 136:585). Maternal age which has been previously shown to be statistically significant [15] was included in the model to adjust its effect. The proposed 2-step procedure and the full model approach were used to analyze data. Table 4 presents results of the analysis.The permutation procedure was handled a bit differently in the model selection step in this analysis: we calculated the maximum information gain ratio at all genomic loci across the genome for each permutation, that is, 742 values for 1 permutation; and the maximum information gain ratios for 20 permutations (a total of 742 × 20 = 14840 values) were collected and used to obtain empirical P-values. This reduces the computational time and allows us to address the multiplicity issue. A cut-off value of *τ *= 0.05 was used in the model selection step because we try to find as many true positives as possible, although the chance that we make the type I error may be slightly inflated when maternal and/or fetal main effects co-exist with the MFGI effect.

**Table 4:** **List of SNPs with maternal-fetal genotype incompatibility effect associated with pPROM at *α *** **=0 *. *** **005**

**Gene**	**Region**	**rs Number**	**P-value**^**1**^	**P-value**^**2**^	**MS**	OR^*^	**95 *% * CI**
*MGP*	promoter	rs1800801	0.0006	0.0404	5	0.4175	[0.2343, 0.7438]
*MMP14*	exon 5	rs2236302	0.0014	0.0051	3	2.8013	[1.6398, 4.7854]
*COL5A2*	exon 48	rs6434312	0.0017	0.0045	10	0.5370	[0.3502, 0.8233]
*ANGPT2*	intron 6	rs2979671	0.0020	0.1450	1	0.2820	[0.0968, 0.8216]
*ANGPT2*	exon 4	rs3020221	0.0020	0.0259	1	0.2826	[0.0947, 0.8434]
*TNFRSF1A*	intron 4	rs1800692	0.0022	0.0968	7	2.1064	[1.3307, 3.3342]
*AQP2*	exon 4	629722653	0.0027	0.0271	2	2.8062	[1.4721, 5.3495]
*CRHR1*	intron 7	rs16940668	0.0038	0.0038	11	1.7365	[1.1605, 2.5986]
*COL1A2*	intron 46	rs13240759	0.0041	0.0041	9	0.5305	[0.3404, 0.8265]
*GJA4*	exon 2	rs1764389	0.0044	0.0044	11	1.6831	[1.1005, 2.5740]
*HLA-E*	exon 3	rs1264457	0.0046	0.6216	8	0.5468	[0.3491, 0.8566]
*IL10*	intron 4	rs5743627	0.0048	0.0048	11	1.3354	[0.8149, 2.1885]
*COL4A2*	intron 33	rs41315048	0.0049	0.0101	10	0.5709	[0.3718, 0.8767]

^1^: P-value obtained using the 2-step approach;

^2^: P-value obtained using the full model approach;

^*^Odds ratio for the MFGI effect;

MS: Model selection result;

OR: Odds ratio;

CI: Confidence interval.

## Results

### Simulation results

**Figure 1:**
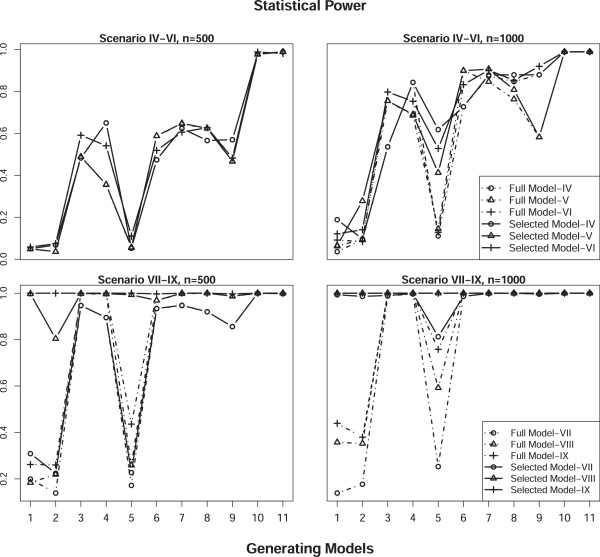
**Statistical power estimates of the proposed model selection approach (solid) and the full model approach (dashed) for Scenarios IV-IX (as given in Table **3**) assuming HWE with sample sizes of 500 (left) and 1000 (right) using 1000 replicates.**

**Figure 2:**
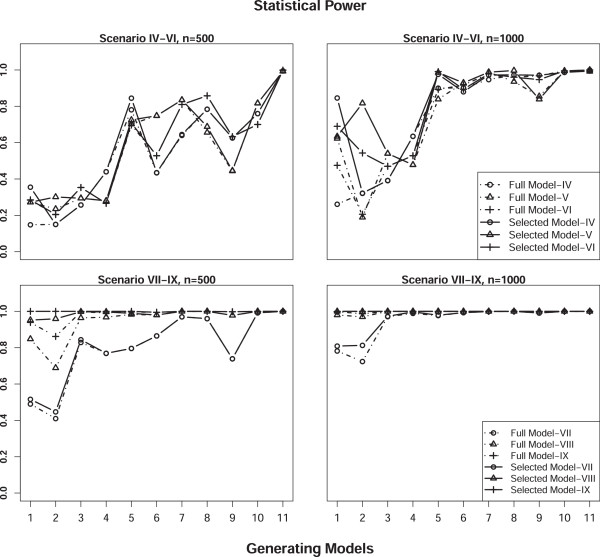
**Statistical power estimates of the proposed model selection approach (solid) and the full model approach (dashed) for Scenarios IV-IX (as given in Table **3**) not assuming HWE with sample sizes of 500 (left) and 1000 (right) using 1000 replicates.**

**Figure 3:**
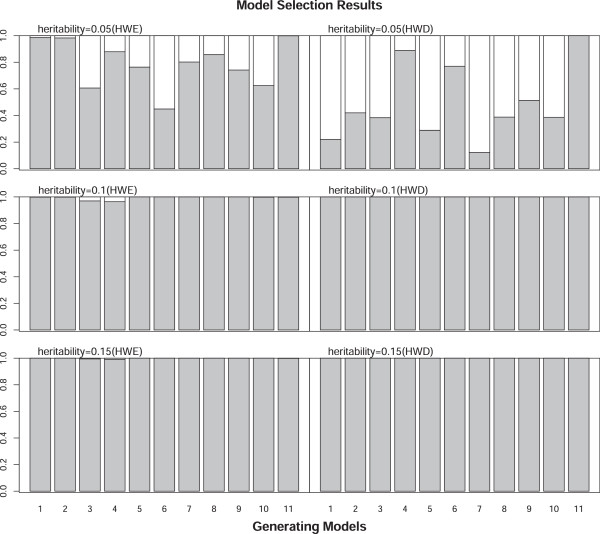
Proportions of the simulations that select the true data generating model (black portion) for scenarios VII-IX (from top to bottom) under HWE (left panel) and HWD (right panel) based on 1000 replicates.

To assess the type I error rate, we simulated the phenotype under the null hypothesis of no MFGI effect. Specifically, data were generated under Scenarios I-III with sample sizes of 500 and 1000. Empirical type I error rates were estimated as the proportion of simulations with P-value less than 0.05 across 11,000 replicates. Overall, the test size was well controlled at the nominal level (0.05) for both approaches under all scenarios we considered. The estimates of type I error rate for the model selection approach relies on the cutoff value *τ* used in the model selection step. According to our simulations, the empirical type I error rate exceeds the nominal level slightly under scenarios II and III, where either maternal or fetal main effect was simulated, when a loose cutoff value of *τ *= 0.05 was used, the obtained empirical type I error rate is around 0.06 (detailed data not shown here). As the cutoff value gets more stringent, the obtained empirical type I error rates approaches to the nominal level. Table 5 presents results of type I error rate obtained with *τ *= 0.0001, which are controlled at the nominal level. The subsequent power estimates were also based on *τ *= 0.0001. As shown in Table 5, the type I error rate for our model selection approach are the same as that for the full model approach under most scenarios. This is because the model selection step almost always chooses the full model (Model 11) when there is no incompatibility effect, leading to the same analysis model for both approaches. There was no significant effect of HWD on type I error. Estimates of the type I error rate for scenarios under HWD are comparable to those for scenarios under HWE.Figures 1 and 2 display statistical power estimates for the proposed model selection approach and the full model approach for testing MFGI. The testing power for our model selection approach was generally higher than that for the full model approach under all the scenarios considered. This improvement was more striking for larger sample sizes. For scenarios that assume HWE, when the MFGI effect was simulated together with maternal and/or fetal main effects (Scenarios IV-VI), our method improved the power, particularly when the true incompatibility model was Model 5. For example, our model selection approach had a power of 0.631 whereas the full model approach only had a power of 0.126 to detect the true MFGI effect when Model 5 was used to generate data with a sample size 1000 under Scenario IV (top right panel of Figure 1). When only the MGFI effect was simulated (Scenarios VII-IX), our model selection approach increased the testing power, especially when the underlying true incompatibility model was Model 1, 2, or 5 (bottom panels of Figure 1). The increase in testing power results from the model selection step, which can choose the true data generating model. The estimated probability of the underlying incompatibility model being selected as the analysis model by our approach approaches 1 with a heritability level of 0.1 or above. With a lower heritability level of 0.05, the estimated probability of selecting true model deceases, especially for scenarios under HWD (right panel of Figure 3). Although improvements in the testing power for HWD scenarios were not as striking as those in HWE scenarios (Figure 2), the performance of our 2-step approach was still better than that of the full model approach.

**Table 5:** Type I error for testing the MFGI effect under simulation Scenarios I-III

		**Scenario I**		**Scenario II**		**Scenario III**	
HWE/D^*^	**Model**	***n *** **= 500**	***n *** **= 1000**	***n *** **= 500**	***n *** **= 1000**	***n *** **= 500**	***n *** **= 1000**
HWE	Full model	0.0566	0.0512	0.0475	0.0540	0.0527	0.0490
	Model selection	0.0566	0.0512	0.0475	0.0540	0.0527	0.0492
HWD	Full model	0.0511	0.0470	0.0492	0.0529	0.0536	0.0477
	Model selection	0.0511	0.0470	0.0492	0.0537	0.0536	0.0483

^*^: HWE: Hardy-Weinberg equilibrium; HWD: Hardy-Weinberg disequilibrium.

### Data analysis results

Table 4 summarizes results of the pPROM data analysis for the 2 approaches. It is evident from the table that our 2-step approach identified MFGIs that could be missed by the full model approach. For example, a P-value of 0.002 was obtained for both SNPs (rs2979671 and rs3020221) in the intron 6 and exon 4 regions of the gene ANGPT2 by using our proposed approach. However, the P-values of 0.1450 and 0.0259 were obtained for SNPs rs2979671 and rs3020221, respectively, by using the full model approach. Model 1 was selected as the incompatibility model for SNP rs2979671 in ANGPT2. SNPs with an odds ratio (OR) less than 1 showed protective effects with the defined genotype incompatibility combinations (Table 4). Here, OR refers to the ratio of odds of developing pPROM in the two risk groups defined by the selected MFGI model. For example, SNP rs2979671 in ANGPT2 had an OR of 0.282, which implies that individuals with the mother offspring paired genotype combination (A/A, G/A) have a lower likelihood than other genotypes of developing pPROM. Such protective effects were also observed for SNPs identified in genes MGP, COL5A2, COL1A2, HLA-E, and COL4A2 (ORs and CIs shown in Table 4).

In comparison, SNPs identified in genes MMP14, TNFRSF1A, AQP2, CRHR1, and GJA4 had OR greater than 1, indicating that a high risk of pPROM is possible with the mother-offspring pairs who have certain genotype incompatibility combinations defined by the corresponding selected incompatibility models. For example, SNP rs2236302 in the exon 5 region of gene MMP14, mother-offspring pairs who have the genotype combination (C/G, C/C) are at higher risk of developing pPROM: 33 of the 104 mothers in the defined “high risk” group developed pPROM whereas only 99 of 611 mothers in the “low-risk” group developed pPROM (OR = 2.8013, 95% CI = [1.6398, 4.7854]). The confidence interval of the OR for SNP rs5743627 in gene IL10 covers 1, indicating that the MFGI effect is not marginally significant. As we are aware of, this is the first analysis that have been done which specifically investigates the genotype incompatibility effect between maternal and fetal gene that underlying pPROM. We believe that our analysis results are helpful for generating hypotheses for future studies or wet lab validations.

## Discussion and conclusions

The importance of maternal-fetal genotype incompatibility in human diseases, particularly in obstetrical complications, was first discussed in the 1990s [38] and has been studied intensively in recent years [16-19,23,24,26]. Most of the currently available statistical methods for identifying MFGI effects fall in the framework of generalized linear regression [20-22,25,30]. Since the underlying MFGI mechanism is unknown and may vary for different genetic variants, it is challenging to appropriately model the incompatibility effect. The complexity largely relies on the underlying competition of 3 sets of genes: the maternally-derived fetal gene, the paternally-derived fetal gene, and the untransmitted maternal gene [39]. Conflict among the 3 sets of genes may result in an incompatibility effect, which may adversely lead to pregnancy complications such as pPROM.

A commonly used approach is to code the incompatibility effect whenever there is a disagreement between maternal and fetal genotypes [30]. However, our simulation studies show that this simple treatment ignores the underlying disease gene action modes and has potential drawbacks. When maternal and/or fetal main effects exist, the method increases the false-positive rates for incompatibility detection. Rather than predefining an incompatibility model, herein, we propose a strategy to select an optimal incompatibility model that captures the underlying disease gene function. A model is selected as a candidate model if its entropy-based measurement is the maximum among all possible incompatibility models via a permutation procedure. The candidate model is then chosen as the analytical model for further statistical tests to assess the incompatibility effect along with the maternal/fetal main genetic effects.

Intuitively, our approach will boost the statistical power by adding a MFGI model selection step. The power gain results from the fact that the true underlying incompatibility model can be selected most of the time with enough samples. We conducted extensive simulation studies, considering the effect of heritability, assumption about HWE, sample size and different disease gene functions. The results indicate that the proposed 2-step strategy works well when the underlying truth is unknown compared with the full model approach. Our approach controls the type I error rate at the nominal level and achieves higher power than the full model approach without performing incompatibility model selection. Our approach does not pose strong assumptions, and its performance is quite consistent under settings such as HWE or HWD, with or without maternal and/or fetal main effects.

We applied the 2-step approach to study maternal-fetal genotype incompatibility effects associated with pPROM and identified several interesting SNPs. Our findings provide clues about the biological mechanism through which MFGI in these genes may have an adverse or protective effect on pPROM. Our results can be used to generate hypotheses for future biological validations to study pathogenesis of pPROM.

Overall, this method can be applied to study the maternal-fetal genotype incompatibility component of obstetrical complications, such as preeclampsia and other human diseases in which maternal and fetal genetic factors interact and increase the risk of disease.

## Competing interests

The authors declare that they have no competing interests.

## Authors’ contributions

SL developed the model, performed the statistical analysis, and drafted the manuscript; YC conceived the idea, participated in the model design and manuscript writing. RR collected the data. All authors read and approved the final manuscript.
